# View on Metformin: Antidiabetic and Pleiotropic Effects, Pharmacokinetics, Side Effects, and Sex-Related Differences

**DOI:** 10.3390/ph17040478

**Published:** 2024-04-08

**Authors:** Guglielmina Froldi

**Affiliations:** Department of Pharmaceutical and Pharmacological Sciences, University of Padova, 35131 Padova, Italy; g.froldi@unipd.it; Tel.: +39-049-8275092

**Keywords:** AMPK, miRNA, antidiabetic drugs, ADRs, sex differences, ADMET, pharmacovigilance, T2DM

## Abstract

Metformin is a synthetic biguanide used as an antidiabetic drug in type 2 diabetes mellitus, achieved by studying the bioactive metabolites of *Galega officinalis* L. It is also used off-label for various other diseases, such as subclinical diabetes, obesity, polycystic ovary syndrome, etc. In addition, metformin is proposed as an add-on therapy for several conditions, including autoimmune diseases, neurodegenerative diseases, and cancer. Although metformin has been used for many decades, it is still the subject of many pharmacodynamic and pharmacokinetic studies in light of its extensive use. Metformin acts at the mitochondrial level by inhibiting the respiratory chain, thus increasing the AMP/ATP ratio and, subsequently, activating the AMP-activated protein kinase. However, several other mechanisms have been proposed, including binding to presenilin enhancer 2, increasing GLP1 release, and modification of microRNA expression. Regarding its pharmacokinetics, after oral administration, metformin is absorbed, distributed, and eliminated, mainly through the renal route, using transporters for cationic solutes, since it exists as an ionic molecule at physiological pH. In this review, particular consideration has been paid to literature data from the last 10 years, deepening the study of clinical trials inherent to new uses of metformin, the differences in effectiveness and safety observed between the sexes, and the unwanted side effects. For this last objective, metformin safety was also evaluated using both VigiBase and EudraVigilance, respectively, the WHO and European databases of the reported adverse drug reactions, to assess the extent of metformin side effects in real-life use.

## 1. Introduction

**Figure 1:**
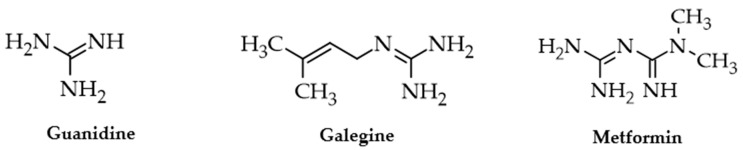
The natural compounds guanidine and galegine recognized in *Galega officinalis* and the synthetic derivative metformin that is mainly used in the treatment of type 2 diabetes mellitus. Chemical structures were drawn using ACD/ChemSketch 2023.2.1 software.

Metformin (1,1-dimethylbiguanide) is a low-molecular-weight compound derived from guanidine, which exists in the human body as a cationic molecule (Figure 1). It was synthesized based on the chemical structure of guanidine derivatives extracted from the European medicinal plant *Galega officinalis* L. (Fabaceae), which has been used for curative purposes since the Middle Ages [1,2]. Various documents attest that as early as the XVIII century, herbal preparations containing the aerial flowering parts of galega were recommended for people affected by persistent thirst and frequent urine emissions, characteristic symptoms of diabetes mellitus (DM) [2]. Subsequently, guanidine and its natural derivative, galegine, were investigated as antidiabetic drugs, demonstrating not only hypoglycemic effects but also some toxicity [2]. In 1922, metformin was first synthesized by Werner and Bell, but it was only later recognized as a hypoglycemic drug [3]. It appears now curious that in the 1950s metformin was used to treat influenza and malaria, considering the antihyperglycemic action a side effect [4,5]. Indeed, only many years later, metformin was approved as an antidiabetic drug in European countries and later in many other states [6]. In 2011, the World Health Organization included metformin among the essential drugs for humanity, and, as such, it is still considered [2,7,8].

Its effectiveness as an antidiabetic drug was recognized worldwide in 1998 thanks to the “United Kingdom Prospective Diabetes Study” (UKPDS 34), which showed that intensive glycemic control with metformin reduced cardiovascular mortality and increased survival of 342 overweight and obese type 2 diabetic subjects to a greater extent compared to sulfonylureas (542 subjects) or insulin (409 subjects) [9]. Afterward, other clinical studies confirmed the antidiabetic efficacy of metformin and its usefulness in reducing cardiovascular complications related to uncontrolled hyperglycemia. Among these, a retrospective study published in 2005 by Johnson et al. demonstrated that treatment with metformin alone, or in combination with sulfonylureas, can reduce the risk of cardiovascular-related non-fatal and fatal events compared to patients treated with a sulfonylurea alone [10]. In 2008, Holman et al. published data from monitoring subjects who had been enrolled in the UKPDS for over 10 years. This study showed that metformin treatment of obese diabetic subjects led to a 21% reduction in diabetes-related conditions, a 33% reduction in myocardial infarction, and a 27% reduction in all-cause mortality [11]. Likewise, the efficacy of the drug in non-obese subjects was confirmed by a retrospective observational study by Ong and colleagues published in 2006, involving normal-weight or overweight subjects [12]. Lastly, in a meta-analysis based on 35 clinical trials, metformin monotherapy (15 trials that included 2424 subjects) showed a reduction in glycated hemoglobin (HbA1c) levels of 1.12% (95% CI 0.92–1.32, *p* < 0.00001) compared to placebo, no treatment, or only diet [13]. 

The effectiveness of metformin in glycemic control and preventing cardiovascular diseases is widely evidenced, however, new antidiabetic drugs have been developed in recent times, offering themselves as possible therapeutic alternatives. Among these, the main contemporary classes in use include the glucagon-like peptide 1 (GLP1) agonists, the dipeptidyl peptidase-4 (DPP4) inhibitors (known as gliptins), and the sodium–glucose cotransporter 2 (SGLT2) inhibitors (known as gliflozins) [14,15]. Even so, metformin remains one of the most prescribed drugs for the treatment of type 2 diabetes mellitus (T2DM) either alone or in association with other antidiabetic drugs [16,17]. This primacy is due to its therapeutic efficacy, reduced serious side effects, such as hypoglycemic crises, and low cost. However, the new guidelines of the European Society of Cardiology indicate that metformin is the first choice drug in patients without overt cardiovascular events, without heart failure (HF), and with preserved kidney function (eGFR ≥ 60 mL/min) [18]. Similarly, in the American Diabetes Association (ADA) Guidelines 2024, metformin no longer appears as the first-choice drug in all antidiabetic treatments, since it has been indicated as a second-line therapy in patients with a high risk for cardiovascular diseases or overt heart and kidney diseases [19,20]. In detail, in diabetic patients with previous cardiovascular events without HF, metformin is considered the first-choice drug, as well as the analogues of GLP1 and SGLT2 inhibitors, while in patients with HF, the first-line drugs are now the inhibitors of SGLT2, while metformin and the GLP1 receptor agonists are the second-line drugs [18]. Therefore, the predominance of metformin is partially weakened by the newly introduced drugs on the pharmaceutical market. However, metformin retains its status for its wide use in T2DM and also for several off-label uses, such as impaired glucose tolerance, obesity, and polycystic ovary syndrome [21,22]. 

The main purpose of this review was to point out the data published in the last 10 years concerning the effectiveness and undesirable effects of metformin in its real-world use, also by using VigiBase and EudraVigilance, respectively, the WHO and European databases of the reported adverse drug reactions (ADRs) [23,24]. Its pharmacokinetics and several off-label clinical uses have also been considered. Attention was paid to the potential differences in efficacy and side effects reported in women compared to men in the clinical use of metformin.

## 2. Results

### 2.1. Pharmacodynamics: Pharmacological Targets

Metformin is an insulin sensitizer as it improves the tissue response to insulin, acting mainly in the liver, muscles, and adipose tissue [25,26]. It decreases glycemia in T2DM subjects by reducing insulin levels, mainly by decreasing liver gluconeogenesis and glycogenolysis [26,27]. Blood glucose levels are reduced both after meal and in fasting conditions, resulting in a decrease in HbA1c, as reported in several clinical trials [11,13]. Its antihyperglycemic effect does not emerge if no insulin secretion occurs [28]. Of interest, metformin treatment is accompanied by a moderate reduction in body weight (3–4 kg) of the subjects and, in the long-term use, by a decrease in cardiovascular risk with favorable effects on vascular endothelium and antioxidant and anti-inflammatory actions [29,30]. A recent study of sex differences of metformin antihyperglycemic effect was evaluated in Chinese subjects treated for 24 and 48 weeks with this drug, showing a similar reduction in HbA1c between females and males [31].

The pharmacological mechanisms by which metformin reduces blood glucose concentration and exerts tissue-protective effects are still under investigation, increasing the emphasis on its role as an interesting multitarget agent [32,33]. Furthermore, several authors have attributed additional therapeutic properties, including antiviral, anti-inflammatory, immunomodulatory, antitumor, and protective effects on lung and pancreatic tissues [34]. In fact, patients with a recent diagnosis of T2DM have a chronic inflammatory state, evidenced by increased levels of IL-6, TNF-α, IL-1β, IL-2 cytokines, and ferritin, which are curtailed after one year of metformin treatment [35].

Evidence obtained from various experimental models suggests that metformin may have different mechanisms of action, particularly when administered at low doses rather than high doses [36,37]. It is generally accepted that metformin acts at the intracellular level by inhibiting mitochondrial respiratory chain complex-1 and regulating cellular energy metabolism, even at supra-pharmacological doses (~1 mM) [27,38,39]. This inhibition can result in an increase in the AMP/ATP ratio and, consequently, the stimulation of AMP-activated protein kinase (AMPK), which plays an important role as a cell regulator of lipid and glucose metabolisms [39,40]. The ability to control cellular metabolism, leading to a situation of relative “energy poverty”, can also explain many of the pleiotropic effects of metformin related to a reduction in energy availability. Its action could be at least in part similar to that induced by intermittent fasting, which can prevent age-related mitochondrial decline and also increase fatty acid oxidation [41,42,43].

Numerous studies in experimental models of diabetic animals and diabetic or obese human subjects have shown that in insulin resistance there is a significant decrease in glucose uptake by glucose transporter 4 (GLUT4) [44,45,46,47,48,49,50]. Effectively, metformin increases insulin-dependent glucose uptake in skeletal muscle and adipose tissue by GLUT4 [51,52].

Of interest, recent research has shown that a molecular target of metformin is presenilin enhancer 2 (PEN2), which interacts with the lysosomal glucose-sensing pathway to activate AMPK, resulting in benefits similar to those generated by fasting [37]. Furthermore, these authors showed by isothermal calorimetry and surface plasmon resonance measurements that the K_D_ for the metformin–PEN2 interaction is 1.7 and 0.15 µmol/L, which are values consistent with the intracellular concentration of the drug reached in vivo in therapeutic use [37]. However, other molecular mechanisms of metformin that are AMPK-independent have been reported to be related to the oxygen consumption rate, tumor suppression, and blocking important pathways such as mTOR [36]. It has also been suggested that the hypoglycemic effect is in part due to a gut-mediated mechanism mediated by increased secretion of GLP1 and peptide YY (PYY) through the intestinal AMPK-dependent pathway [53]. Furthermore, it has also been reported that the increase in GLP1 is due to an inhibition of the enzyme DPP4, which can also regulate the peptide YY level, causing a reduction in food intake and an increase in satiety [54,55,56]. Recently, Wang and colleagues have highlighted the existence of a DPP4 enzyme produced by the intestinal microbiota that metabolizes human GLP1, which is potentially active in cases of impaired intestinal permeability due to inflammation, as occurs in DM [57]. Moreover, several authors have described that metformin changes the intestinal microbiota while also decreasing glucose absorption [57,58,59,60,61,62]. Therefore, the hypoglycemic action of metformin may also be mediated by intestinal-dependent mechanisms, which could vary depending on microbiota phenotype [63]. Inhibition of IL-6 mediated signaling by decreasing the expression of its receptor at the transcription level via AMPK, mTOR, and miR-34a is one of the newer mechanisms that suggest metformin as a potential treatment for multiple myeloma [64]. MicroRNAs (miRNAs or miR) are small, single-stranded, non-protein-coding RNAs that regulate gene expression post-transcriptionally by degrading target mRNA when there is a perfect match or by inhibiting translation when there is imperfect complementarity [65]. Different miRNAs can promote or inhibit DM complications [66]. Several authors have explored circulating miRNAs in T2DM compared to healthy subjects, suggesting that specific miRNAs might serve as biomarkers for early diagnosis of T2DM [66,67,68]. Various studies have also shown changes in miRNA expression in subjects treated with metformin [69,70,71]. A study with 47 patients (20 women and 27 men) with T2DM showed that a total of 13 miRNAs, such as miR-let-7e-5p, let-7f-5p, miR-21-5p, miR-24-3p, miR-26b-5p, miR-126-5p, miR-129-5p, miR-130b-3p, miR-146a-5p, miR-148a-3p, miR-152-3p, miR-194-5p, and miR-99a-5p, were significantly negatively regulated after a three-month treatment with metformin, while the other 73 miRNAs were not altered [69]. Specifically, metformin significantly altered the expression of miR-148a-3p and miR-194-5p (*p* < 0.007), potentially affecting the NF-*k*B and Wnt signaling pathways [69]. Furthermore, metformin can up-regulate miRNA-185-5p expression to suppress glucose-6-phosphatase (G6Pase) and inhibit liver gluconeogenesis, reducing fasting blood glucose levels [72]. Although there are various studies aimed at identifying the effects of metformin on miRNA expression, the currently available data do not allow for definite conclusions, thus, further investigations are required involving a larger number of subjects treated with fixed doses of the drug.

### 2.2. Pharmacokinetics: Absorption, Distribution, Metabolism, and Excretion (ADME)

**Figure 2:**
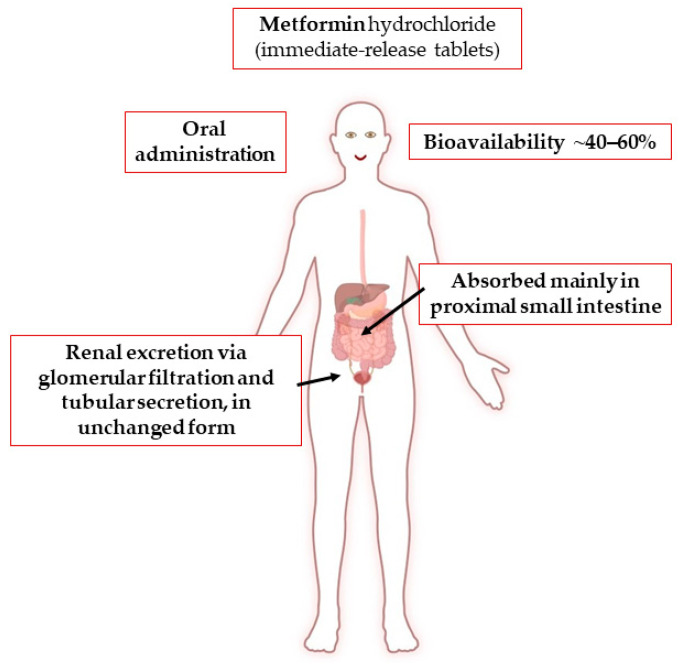
Absorption, distribution, metabolism, and excretion of metformin. Created with BioRender software.

Metformin hydrochloride is administered orally along with meals in doses ranging from 500 to 3000 mg per day, subdivided into two or three times (Figure 2). It has a dose-dependent antihyperglycemic effect [73,74,75]. It is also prescribed for children aged at least 10 years. The pharmacokinetics of metformin have been reported to be similar in women and men, considering the weight differences between the sexes [76].

To limit the occurrence of gastric and intestinal disorders, namely, nausea and diarrhea, a low initial dose (500 mg, once or twice per day) is administered, and, after 10–15 days, the dose is increased to obtain the target glycemic control (maintenance dose) [15,77]. Furthermore, it has been observed that taking it during or immediately after meals can help reduce the typical gastrointestinal side effects [77]. More recently, various types of delayed-release formulations have been marketed that allow a single evening administration and, potentially, even fewer gastrointestinal disorders [78,79,80]. However, comparative clinical studies between immediate-release and controlled-release formulations are rather limited and difficult to interpret for the different dosages used, the treatment period, and the different number of subjects enrolled [78,81].

Due to its basic hydrophilic nature, the absorption of metformin by simple diffusion through the intestinal mucosa would be extremely low and unsuitable for therapeutic use. In fact, absorption is dose-dependent and occurs through an active and saturable process by means of membrane proteins (transporters) [82]. Thus, after oral administration, about 40–60% of metformin is absorbed primarily in the proximal small intestine (duodenum) through the plasma membrane monoamine transporter (PMAT or SLC29A4) and the organic cation transporters 1 and 3 (OCT1 and 3 or SLC22A1 and 3) [82,83,84]. The drug is eliminated through urine via glomerular filtration and tubular secretion, without enzymatic biotransformation, in its unchanged form [85,86]. A fecal excretion of an unabsorbed fraction (20–30%) is reported [85].

Since metformin exists mostly as a cation in the human organism at the physiological pH, the distribution and renal excretion are also mediated by endogenous transporters, such as OCT1, 2, and 3; PMAT; multidrug and toxin extrusion transporters 1 and 2 (MATE 1 and 2 or SLC47A1 and 2); carnitine/organic cation transporter (OCTN1or SLC22A4); serotonin reuptake transporter (SERT); and thiamine transporter 2 (THTR2 or SLC19A3) [83,87]. Mainly, the OCT1 transporter is expressed at the intestinal level, controlling oral absorption, while OCT1 and 2 regulate renal clearance, respectively, at the luminal and basolateral sides of the proximal and distal tubules [88]. A more detailed description of the transporters involved in metformin life in the human organism is provided in previous reviews [83,88,89]. As is known, the protein transporters involved are expressed differently in human tissues and have high genetic polymorphism, generating wide inter-individual variability in metformin pharmacokinetics, explaining the range of dosage and different antidiabetic effectiveness [87,88,90]. The detected C_max_ is around 1 µg/mL, and, in general, the optimal plasma concentration should be less than 2 µg/mL, whereas concentrations greater than 5 µg/mL are considered unsafe due to the increased risk of severe adverse effects, such as lactic acidosis [87,91,92]. However, several authors reported blood concentrations of up to 80 µg/mL in subjects treated with metformin, showing high variability in the pharmacokinetics of this drug [93]. Aging is known to lead to a progressive decline in renal function, which can result in an increase in metformin blood concentration that may require a dose reduction [94]. A recent meta-analysis of 14 clinical trials (408 subjects) shows that food intake reduces C_max_ by 40% and delays it by ~29%, decreasing the area under the curve by ~28% [95]. In particular, a diet rich in fat and high in calories can significantly decrease the amount and rate of absorption [95]. Metformin binds marginally to plasma proteins, while it is found within erythrocytes, which form a secondary distribution compartment [82,85,93,96]. The apparent volume of distribution is between 63 and 276 L, while the terminal half-life (t_1/2_) is 4.0–8.7 h [97,98]. Metformin can cross the human placenta and is detectable in small amounts in breast milk with a milk-to-plasma ratio between 0.35 and 0.71 [99]. Its renal clearance is ~510 ± 120 mL/min, and its half-life is approximately 5 h [82]. When renal function is impaired, renal clearance decreases proportionally with creatinine clearance, thus prolonging the half-life and increasing the plasma concentration. For this, metformin use is contraindicated when the estimated glomerular filtration rate (eGFR) is less than 30 mL/min/1.73 m^2^ [100].

### 2.3. Metformin and Vascular Diseases

The macro- and microvascular complications caused by hyperglycemia are widely described in the literature and are also identified as panvascular diabetic disease (PVDD) [101,102,103]. In fact, macro- and microvascular complications can coexist, appearing in specific organ damage according to the characteristics of diabetic patients and resulting in several different diseases, such as heart failure, stroke, peripheral vascular disease, chronic kidney disease, diabetic retinopathy, and autonomic neuropathy [104,105,106]. Metformin, in addition to its proven hypoglycemic action, has the potential to reduce hyperinsulinemia, which is a factor that can worsen metabolic disorders such as hyperlipidemia and endothelial dysfunction [107]. This may provide greater cardiovascular protection than insulin secretagogues such as sulfonylureas and glinides, which raise insulin levels [108,109]. The “2022 Chinese Expert Consensus on Risk Assessment and Management of Panvascular Disease in Patients with T2DM” recommends metformin alone, or in combination with GLP1 receptor agonists or SGLT2 inhibitors, as a first-line hypoglycemic agent in the absence of explicit contraindications [110]. Moreover, the ADA Guidelines 2024 indicate the role of GLP1 agonists and SGLT2 inhibitors in the treatment of atherosclerotic cardiovascular diseases, including peripheral artery disease (PAD) [20].

Several in vitro and in vivo studies have shown that metformin plays a protective role against endothelial dysfunction, protecting vasodilation and decreasing inflammation and oxidative damage [111,112,113,114]. Numerous studies have found that women are more susceptible to PAD than men, and women affected by diabetes have an increased risk of developing symptomatic PAD [115,116,117]. Furthermore, patients with DM and PAD are more likely to develop ischemic ulcers or gangrene, also leading to “diabetic foot”, than non-diabetic subjects [118,119]. Notably, poor glycemic control is an independent risk factor for PAD [120]. It has recently been observed that the prevalence of PAD is the same in both sexes in high-income countries, while it is more recurrent in women in low- and middle-income countries, especially in younger subjects [121]. Furthermore, DM causes higher all-cause mortality in women than in men due to cardiovascular diseases with more manifest endothelial dysfunction than diabetic males [122,123]. A few clinical studies have been planned to evaluate the efficacy of metformin in PAD, and enrolled subjects are generally treated with other drugs, such as antiplatelets, statins, and antihypertensives. For this reason, the effective role of this drug in the progression of PAD is still unclear. In patients undergoing revascularization for chronic ischemia of the limbs, Khan and colleagues showed that subjects treated with metformin (*n* = 147) had a significant improvement in survival at 60 months compared to those treated with insulin (*n* = 216) or other oral antihyperglycemic drugs (*n* = 196). However, unfortunately, there was no significant improvement in patency rates or limb salvage after endovascular reperfusion interventions [124]. A prospective study that enrolled 100 T2DM subjects with PAD after a 12-month follow-up showed the effectiveness of metformin in reducing adverse limb events, suggesting the usefulness of the drug in these patients [125]. Therefore, to determine whether metformin can play a role in symptomatic PAD, regardless of antidiabetic action, a prospective study, compared to placebo, is ongoing in non-diabetic subjects with intermittent claudication, called “Metformin BenefIts Lower Extremities with Intermittent Claudication” (MOBILE IC trial) [126]. The expected date for a first analysis of the results has been set for 2026 [108].

### 2.4. Metformin and Brain

Metformin can cross the blood–brain barrier and reach a significant concentration in the central nervous system (CNS), causing pleiotropic effects [127]. Although the data obtained are not always consistent, several studies have suggested the use of metformin in various diseases that affect SNC [128,129]. In male and female mice with spinal cord injuries, 200 mg/kg metformin administered subcutaneously for 14 days increased oligodendrogenesis in both sexes [130]. Additionally, it increased the activation of neural stem and progenitor cells in females and reduced the activation of microglia in males [130]. The neurogenesis and oligodendrogenesis induced by metformin were caused by its action on atypical protein kinase C (aPKC)-mediated phosphorylation of the CREB-binding protein (CBP) [131,132,133]. Other authors also suggested that metformin has anti-inflammatory effects in injured brains, reducing microglia activation via the NF-κB-MAPK signaling pathway [134]. An additional molecular pathway has been reported suggesting activation of Ser/Thr kinase partitioning defective 1 (Par1)/MARK, which is a downstream target of tumor suppressor kinase B1 (LKB1) [135].

Cerebral amyloid angiopathy (CAA) often leads to senile dementia and cerebral lobar hemorrhages and is commonly identified in subjects with Alzheimer’s disease (AD) [136]. Numerous epidemiological studies have demonstrated that individuals with T2DM have a significantly higher probability of developing AD [137,138]. In APP23-*ob*/*ob* mice, a model of CAA and T2DM, metformin (350 mg/kg daily) administered for about 14 months reduced the number of cerebral blood vessels with amyloid β deposits, increasing the insulin-degrading enzyme (insulysin) [136]. However, there are conflicting data on the development of the effect of metformin on the amyloidosis typical of AD. A protective effect due to a decrease in amyloid secretion, generally described as an AMPK-independent pathway, has been reported [139]. However, an increase in amyloid-β formation with higher doses via AMPK activation has also been described, which may exacerbate the pathogenesis of AD [140,141,142].

The population-based “Singapore Longitudinal Aging Study”, which evaluated 365 subjects with T2DM, aged 55 years and older, who were followed for at least 4 years and treated with metformin compared to placebo, showed a significant inverse association with cognitive impairment in cross-sectional and longitudinal analyses, reporting significant protection after 6 years of metformin therapy [143]. A crossover double-blind RCT was carried out on 24 survivors of pediatric brain tumors who received cranial radiation [144]. With the idea that metformin can increase NPCs and repair brain damage, the authors administered metformin (1000 mg/m^2^ orally, twice daily for 12 weeks), showing enhanced performance in declarative and working memory tests [144]. Given the small number of enrolled subjects, even if the results obtained are promising, they certainly need to be validated in a larger number of subjects through further RCTs. It should be noted that a recent meta-analysis of 19 RCTs that explored the risk of cognitive dysfunction, dementia, AD, or Parkinson’s disease (PD), considering a total of 285,966 subjects, revealed that exposure to metformin used for antidiabetic treatment did not reduce the incidence of degenerative CNS diseases (OR 1.04, 95% Cl 0.92 to 1.17) and, regrettably, significantly increased the frequency of Parkinson’s disease (OR 1.66, 95% CI 1.14 to 2.42) compared to non-metformin users [145].

Overall, clinical data on the effects of metformin on preventing and treating cognitive and, in general, neurodegenerative CNS diseases are still fragmentary and inconsistent. Consequently, no definitive conclusions can be drawn.

### 2.5. Metformin and Cancer

It is generally recognized that DM increases the risk of various types of cancer [146,147]. A recent prospective longitudinal cohort study with 428,568 newly diagnosed T2DM subjects, showed that the risk for all-cause cancer was approximately 10% higher in diabetic patients, and the relative risk was higher when T2DM was diagnosed at a younger age [148]. Recently, retrospective studies have shown that T2DM subjects taking metformin display a lower risk of cancer than a healthy population or diabetic patients never treated with metformin [149,150,151]. Several RCTs assessed the impact of metformin on mortality in diabetic patients with colorectal cancer (CRC), showing that it can reduce all-cause mortality and cancer-specific mortality [152,153,154]. However, not all investigations are in agreement; in fact, some have not found a significant protective association between metformin and survival in patients with CRC [155,156].

A recent meta-analysis that considered 8 cohort studies from 2012–2019 showed that CRC subjects with T2DM treated with metformin had a lower overall mortality, even not specific-cause mortality, than CRC patients with T2DM who did not receive metformin [157]. Of interest, this study evidenced a lower mortality in women among CRC patients with T2DM using metformin than in those who did not receive it [157]. Previously, a significant reduction in sex-related incidence was revealed in women treated with low doses of metformin in a prospective cohort study from Taiwan [158]. A meta-analysis based on 37 studies for a total of more than 1.5 million subjects showed that metformin reduces the incidence of cancer in the liver (−78%), pancreas (−46%), colon (−23%), and breast (−6%), as well as mortality from liver and breast cancer [159]. The marked reduction in the incidence of certain types of cancer, such as those of the liver and pancreas, may be explained by the known fact that insulin resistance plays a pivotal role in their growth [160]. Of interest, a large meta-analysis of 121 cohorts, including more than 19 million individuals with about one million all-site cancer events, showed that T2DM is associated with an additional risk of all-site cancer of 6% higher in women than in men [161]. In more detail, DM caused a significantly higher relative risk in women than men for oral, stomach, and kidney cancers and leukemia, whereas the opposite was observed for liver cancer [161].

In general, several systematic reviews and meta-analyses gave comparable results, but with differences in the type of cancer treated, the time and dose of exposure to metformin, the presence of concomitant treatments, etc.; thus, there remains the need to study the real usefulness of metformin in cancer patients regardless of its use as an antidiabetic drug.

### 2.6. Metformin and COVID-19

Recent studies reported that patients with DM have a higher risk of death and a higher probability of being hospitalized with Severe Acute Respiratory Syndrome CoronaVirus-2 (SARS-CoV-2) than those without diabetes [162,163,164]. A recent systematic review revealed that diabetic patients experienced worse clinical outcomes during the COVID-19 pandemic, with an increase in all-cause mortality and diabetes-related mortality; in particular, clinical outcomes worsened in women, children, and individuals of ethnic minorities [165].

Metformin has given promising results in diabetic subjects with Coronavirus Disease 2019 (COVID-19) because, in addition to its hypoglycemic action, it also has antiviral, anti-inflammatory, and immunomodulatory properties [166,167]. Epidemiological data collected from T2DM subjects treated with metformin for glycemic control during the course of COVID-19 revealed a significant reduction in the mortality rate related to the viral disease by a factor of ~3 times [168]. A meta-analysis of 10,233 subjects confirmed the association between metformin use and mortality reduction after adjustment for various comorbidities such as obesity, hypertension, cardiovascular disease, and kidney disease [169]. Similarly, an observational cohort study with data from T2DM individuals reported a significantly lower risk of COVID-19-related mortality in patients treated with metformin [170]. Another study suggested the existence of differences between female and male subjects with respect to the outcomes of severe SARS-CoV-2 syndrome in T2DM subjects treated with metformin [171]. Indeed, retrospectively analyzing the cohort of diabetic or obese women (BMI ≥ 30 kg/m^2^) infected with the virus showed a reduction in the severity of the disease and in the mortality rate. The advantage was not proven in the male cohort [171]. Furthermore, a second retrospective cohort study described that metformin use decreased the onset of acute respiratory distress syndrome in women with DMT2 and COVID-19 [172].

One aspect of metformin therapy that has raised concern, especially in the case of severe SARS-CoV-2 infection, is the risk of metabolic acidosis, since there is a general consensus that this drug has a higher risk of lactic acidosis compared to other antidiabetic agents [173]. However, despite this alarm, no increase in mortality from COVID-19 has been observed among metformin-treated individuals [174]. Otherwise, the authors discovered a significant association between metformin use and decreased heart failure and inflammation damage. In particular, metabolic acidosis was observed in patients taking high doses of the drug, with an impairment of renal function and a worsening of the clinical progression of COVID-19 [174]. Although it is one of the most commonly prescribed antidiabetic drugs due to its effectiveness in DM, even for those infected with the SARS-CoV-2 virus, ongoing monitoring is recommended for patients with impaired renal function to accurately determine appropriate therapeutic dosages [175,176]. The discontinuation of metformin is recommended if the eGFR drops below 30 mL/min/1.73 m^2^, as indicated in patients not affected by COVID-19. Otherwise, the patient would be exposed to excessively high blood concentrations that can cause metabolic acidosis. For this, more attention should be paid to individuals affected by severe COVID-19, among whom a reduced renal function is noted in the various acute sequelae [177,178].

### 2.7. Metformin and Weight Control

Overweight and obesity in diabetic subjects increase the risk of many cardiovascular diseases and even cancer [179,180]. Obese individuals are believed to have a compromised intestinal barrier, leading to impaired intestinal permeability [181]. Reduced body weight has been found to improve intestinal conditions by restoring the selectivity of the intestinal barrier [182,183]. A moderate low-calorie diet together with increased physical activity after 2 and 12 months improved enteral permeability by decreasing chemerin and lipopolysaccharide-binding protein (LBP) levels in adolescents with abdominal obesity [184]. Assuming that body weight reduction can produce similar results regardless of the method used, metformin has been studied in a group of overweight or obese subjects in the “Survivorship Promotion In Reducing IGF-1 Trial” (SPIRIT) [185]. Contrary to expectations and unlike the diet intervention, 6 and 12 months after the drug treatment, metformin did not decrease the level of blood LBP despite achieving a similar degree of weight loss (3%) [185].

Both metformin and gliflozins have been shown to reduce hyperglycemia and HbA1c and are being approved for the treatment of T2DM [186,187]. Gliflozins act as SGLT2 inhibitors by causing glucose urinary excretion in the proximal tubules, decreasing hyperglycemia and body weight [188,189,190,191]. A sub-analysis of a prospective, multicenter, open-label RCT has considered 29 elderly T2DM subjects, receiving sitagliptin as the base antidiabetic treatment, who were treated with either ipragliflozin (50 mg daily) or metformin (500 mg daily), showing that after 24 weeks, the visceral adiposity index decreased with each one [192]. Furthermore, the recent post-hoc analysis compared the metabolomic changes associated with 50 mg ipragliflozin versus 1000 mg metformin daily for 24 weeks in 30 diabetic subjects receiving sitagliptin as basal treatment, showing that the reduction in the visceral fat area was higher with ipragliflozin (−19.8%) than with metformin (−2.5%, *p* = 0.002), as well as for waist circumference and body weight [193]. In general, the metabolomic profile of several biomarkers was different between the metformin and ipragliflozin groups. Briefly, with metformin, a moderate reduction in LDL was highlighted, and the levels of citrulline, octanoic acid, indole-3-acetaldehyde, and hexanoic acid were also reduced [193]. Whereas, hypotaurine, methionine, methyl-2-oxovaleric acid, 3-nitrotyrosine, and cyclohexylamine levels increased [193]. Furthermore, ipragliflozin treatment showed a significant increase in hematocrit and a greater increase in severe liver steatosis compared to metformin treatment [193].

A new possibility of metformin use is for weight control in patients treated with antipsychotics, which generally cause an unwanted increase in weight with higher cardiovascular risk. A recent review that considered 5 clinical trials in 227 participants suggests that metformin may be effective in preventing weight gain caused by antipsychotic drugs [194]. However, there were no adequate clinical trials, and the number of patients enrolled was relatively low. Therefore, additional trials are necessary to validate this use.

### 2.8. Metformin and Polycystic Ovary Syndrome

Polycystic ovary syndrome (PCOS) is among the most common reproductive endocrine disorders, affecting approximately 4 to 20% of women in their reproductive years [195,196,197,198]. The “International Evidence-based Guidelines for the Assessment and Management” of PCOS recommended that adult women should be treated with combined oral contraceptive pills (COCP) using low-dose preparations, which is an off-label pill use, in the presence of hyperandrogenism and/or irregular menstrual cycles [199]. Metformin is recommended to treat metabolic disorders that are common in PCSO, either as a standalone treatment or as an additional therapy [199,200]. This endorsement is based on the fact that nearly half of women with PCOS are overweight or obese and may have impaired glucose tolerance, hyperinsulinemia, or overt T2DM [201,202]. It is important to note that metformin aids in regulating menstrual cycles and improving fertility [196,199].

Recently, an open-label prospective RCT compared a combination of metformin (1000 mg twice daily) and canagliflozin (100 mg daily), an SGLT2 inhibitor, with metformin alone for a 3-month treatment in 51 overweight or obese non-diabetic PCOS women aged 18–40 years [203]. Metformin alone showed similar effects to combination therapy on menstrual frequency, weight control, hyperandrogenemia, and insulin resistance, although a greater reduction in testosterone levels and total area under curves of glucose and insulin was observed with combination therapy [203]. Another RCT involving 52 overweight participants compared the efficacy of metformin alone (1000 mg twice daily) with a combination of metformin and liraglutide (metformin taken twice daily, orally, along with liraglutide 1.2 mg once daily, subcutaneously). The results indicated comparable improvements in menstrual cycles, anthropometric measures, and glucose metabolism after the 12-week treatment period [204]. However, the combination of metformin and liraglutide exhibited superior efficacy in improving reproductive abnormalities and hyperandrogenemia, possibly modulating the hypothalamic–pituitary–ovarian axis [204].

Recently, Greff et al. conducted a systematic review of selected 26 RCTs, including 1691 women with PCOS, comparing the efficacy and safety of inositol compared to metformin or placebo treatments [205]. The authors demonstrated that inositol exhibited efficacy comparable to metformin in terms of weight loss, cycle normalization, and testosterone levels. In addition, improvements in hyperinsulinemia and carbohydrate metabolism were observed compared to the placebo group, suggesting that inositol may not be inferior to metformin treatment regarding glucose control [205]. A meta-analysis of 9 RCTs with a total of 612 subjects showed that the administration of myo-inositol (2000 to 4000 mg daily) compared to metformin (1500 to 2550 mg daily) could be more effective in lowering triglycerides and cause fewer side effects of metformin [206].

A very recent meta-analysis of 46 RCTs has been performed as a 2023 update of the “International Evidence-based Guidelines for the Assessment and Management of PCOS”, highlighting that metformin is superior to hormonal treatment in controlling the blood sugar, overweight, and lipid profile of PCSO subjects [207,208]. Therefore, metformin is considered a second-line treatment in PCOS that offers benefits for the management of menstrual and ovulatory disorders, hirsutism, and metabolic and cardiovascular disorders [209,210].

An RCT of 65 women with PCOS treated with COCP (150 mg desogestrel + 30 μg ethinylestradiol) alone, metformin (2000 mg daily) plus COCP, or metformin alone for 12 months showed significant differences in miRNA levels between the metformin-treated groups and those receiving COCP alone [211]. Among 22 selected miRNAs known to be related to PCOS, lipid, and glucose disorders, miR-122, mi-R29a, and miR-223 decreased significantly, suggesting their role in metformin protection in PCOS subjects [211].

However, not all the data reported in the literature are consistent. A systematic review that included 44 RCTs (2253 women), with 39 studies in adult women (2047 subjects) and 5 in adolescent women (206 subjects), found no significant evidence that metformin treatment alone is more effective than COCP, or vice versa, or than combined COCP and metformin administration [212]. New clinical studies stratified by age and symptoms must be performed to validate the therapeutic use of metformin in women with PCOS.

### 2.9. Metformin and Thyroid Disorders

Endocrine autoimmune diseases account for most thyroid diseases (TDs), which have a high prevalence in the global population (5%), occurring at least 5–8 times more frequently in females than males [213,214,215,216]. TDs are distinguished by either an increase in thyroid activity, that is, Graves’ disease, or a decrease in activity, that is, Hashimoto’s thyroiditis. Autoimmune thyroid diseases can lead to altered glucose metabolism, thereby heightening the risk of developing DM [217,218].

To investigate the influence of metformin on thyroid function, a clinical study has recruited 19 healthy male volunteers, showing that metformin does not alter iodine uptake in the thyroid gland [219]. However, various clinical studies performed in hypothyroid subjects have shown that metformin reduces thyrotropin (TSH) levels even if the mechanism involved is not fully understood [220,221]. A prospective study considered 100 euthyroid subjects (68 females and 32 males) with insulin resistance treated with metformin (1700 mg daily) for six months, resulting in a significant decrease in body weight, insulin resistance, and TSH level and, in addition, a reduction in thyroid volume and the size of thyroid nodules [222]. The authors suggested that hyperinsulinemia per se may promote an increase in thyroid size and the presence of nodules that may improve through the use of metformin, which decreases hyperinsulinemia. In fact, insulin, through IGF-1 signaling, is known to modulate thyroid gene expression, as an additional factor in thyrocyte proliferation and the differentiation of thyrocytes. In addition, metformin can cause antiproliferative effects by suppressing the activity of the mammalian target of rapamycin (mTOR) [223,224].

A systematic review and meta-analysis that considered about 150 subjects showed that metformin moderately reduces the size of thyroid nodules and decreases the level of TSH and insulin resistance, even if it does not change the size of the thyroid gland [225]. These results are in agreement with those obtained in a previous meta-analysis that enrolled 240 patients with benign thyroid nodules and insulin resistance [226].

### 2.10. Adverse Drug Reactions: The Benefit/Risk Ratio

Subjects taking metformin, especially in the first one–two weeks of intake, frequently experience nausea, vomiting, and abdominal discomfort, with a prevalence of 8 to 21% [206]. Several investigations suggested that the gastrointestinal (GI) side effects are mitigated by starting therapy with low doses and taking the drug during meals [227]. Moreover, the administration of controlled and gradual release formulations may be useful in reducing GI discomforts [228,229]. To explain these gut effects, metformin has been suggested to cause 5-hydroxytryptamine release at the intestinal level, then act by a mechanism independent of 5-HT_3_ receptors [230]. In fact, several authors, through in vitro and in vivo investigations, showed that GI disorders may depend on the genotype of SERT and OCT1 transporters [231,232]. Furthermore, metformin can also increase intestinal release of GLP1, which is known to slow gastrointestinal transit and reduce appetite (anorectic effect) [54].

Indeed, various clinical studies have shown that about 34% of subjects who begin metformin treatment undergo at least one ADR during a 1-year follow-up period [107,233]. It was also observed that the proportion of undesirable effects was higher among women than among men [233,234]. In particular, the longitudinal study “Lareb Intensive Monitoring (LIM) program” of the Dutch National Pharmacovigilance Center Lareb has included 1712 diabetic subjects with an average age of 58 years, of which 40.9% were women, who were followed for 12 months by an online questionnaire [233]. The study showed that women reported ADRs more frequently in the first period of metformin treatment (at 2 and 6 weeks of starting therapy) than men, while in the following period the recurrence of the reported adverse effects was the same between the two sexes [233]. These studies highlighted the usefulness of carefully evaluating the beginning dose of metformin, as the dose required for antidiabetic treatment in women is generally lower than in men. Women who receive a lower initial dose than men could reduce the appearance of ADRs by improving their adherence to T2DM treatment.

Other side effects commonly reported are headache, dizziness, fatigue, pruritus, and dysgeusia [233,234]. One of the most feared adverse reactions to metformin treatment is lactic acidosis, which is a rare event with an incidence of 1 in 30,000 patients but can lead to fatal outcomes [91,235]. Generally, the observed cases occurred in patients who received high doses and/or had severe hepatic and renal impairment, old age, and alcoholism [236,237]. In particular, the risk of ADRs increases in patients receiving polytherapy because of drug–drug interactions. Various reviews are available in the literature regarding this topic [238,239,240,241,242].

Prolonged use of metformin is linked to the reduced vitamin B12 and elevated levels of homocysteine and methylmalonic acid, which can lead to anemia and diabetic peripheral neuropathy [243,244,245]. Patients with DM2 were studied in an RCT to examine the effects of prolonged use of metformin, observing that vitamin B12 levels were significantly lower in the metformin group (231 vs. 486 pmol/L; *p* < 0.001), with a frank deficiency in 18 patients (31%) compared to 2 subjects (3%) of the untreated group [246]. It should be noted that the metformin dose is inversely correlated with the amount of serum vitamin and clinically more severe peripheral neuropathy [246]. These data highlight the importance of monitoring the level of vitamin B12 in patients undergoing prolonged metformin therapy and also in subjects who, due to dietary choices, such as vegans, have an additional risk of vitamin B12 deficiency [247]. Recently, a dose-dependent correlation between long-term metformin use and peripheral neuropathy has been confirmed in a clinical trial in Chinese subjects with T2DM [248]. In addition, vitamin B12 deficiency has also been associated with the worsening of various other CNS diseases and, in particular, of PD, explaining the potential correlation between metformin treatment and the worsening of Parkinson’s disease [145,249].

**Figure 3:**
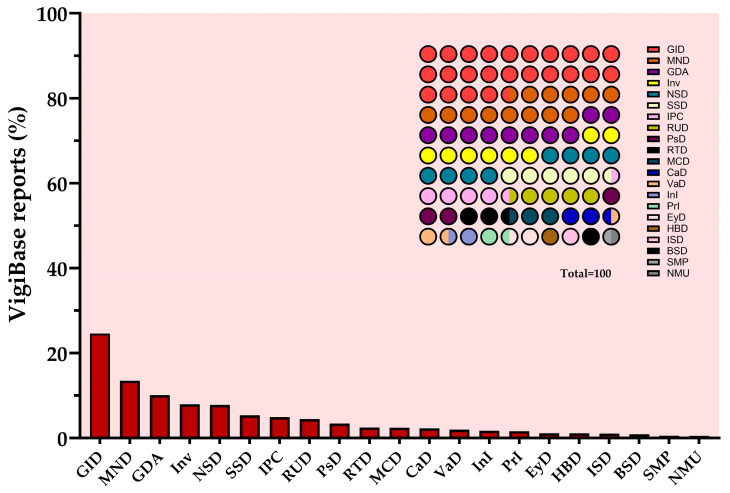
VigiBase reports of ADRs potentially caused by metformin in real-word therapy. GID = gastrointestinal disorders; MND = metabolism and nutrition disorders; GDA = general disorders and administration side conditions; Inv = investigations; NSD = nervous system disorder; SSD = skin and subcutaneous tissue disorders; IPC = injury, poisoning, and procedural complications; RUD = renal and urinary disorders; PsD = psychiatric disorders; RTD = respiratory, thoracic, and mediastinal disorders; MCD = musculoskeletal and connective tissue disorders; CaD = cardiac disorders; VaD = vascular disorders; InI = infections and infestations; PrI = product issues; EyD = eye disorders; HBD = hepatobiliary disorders; ISD = immune system disorders; BSD = blood and lymphatic system disorders; SMP = surgical and medical procedures; NMU = neoplasms benign, malignant, and unspecified. Created with GraphPad Prism 10.1.2 software.

It can be expected that the widespread use of metformin in therapy will be accompanied by increased reports of side effects worldwide. To verify this point, the current search using the VigiBase resource shows a total of 120,083 reports of ADRs since the 1990s until 10 January 2024 [250]. The majority of spontaneous side effects occurred in the Americas (41%), Asia (31%), and Europe (24%) and were mainly distributed in patients aged 45–64 years (35%) of female sex (56%) [250]. Figure 3 shows the side effects found in the database in decreasing order of frequency; among the most recurrent ADRs, those in the GI (24%), in agreement with the data from clinical trials. Some cases of lactic acidosis and hypoglycemia (metabolism and nutrition disorders), drug ineffectiveness, and fatigue (general disorders) were also attested (Figure 3). The ADR ratio (ADRs in women/ADRs in men) obtained from records in VigiBase is 1.43, suggesting a higher risk of ADRs in women than in men. In agreement, several studies have shown that women are more likely to experience adverse reactions caused by hypoglycemic drugs, including metformin [233,234,251]. At least in part, the failure to adjust the dose of metformin administered to women may explain the increased appearance of side effects compared to men.

ADR research has also been implemented in EudraVigilance, the European database of suspected ADR reports, to more closely identify reports in Europe and then make a comparison between the two resources. In EudraVigilance, by a search up to 27 January 2024, a total of 42,557 cases were found, of which 16,436 were in subjects aged 18–64 years, and 16,248 in older subjects aged 65–85 years. Among the reports, those related to women were the majority, equal to 21,635 (50.8%). Most cases were reported in France (25%), followed by Italy (16%), and then Germany (14%). Unlike VigiBase data, the most frequently reported ADR group was “Metabolic and nutritional disorders” (Figure S2). Considering the smaller size of the European database, the information from both databases is generally comparable.

Very few clinical studies have reported sex-related differences in efficacy and safety in the therapeutic use of metformin. The main differences identified in this review are summarized in Table 1.

## 3. Materials and Methods

In this review, publications identified using the PubMed database were considered by searching up to the end of February 2024. The search was restricted to items published in English in the last 10 years by using the keywords “metformin” and “gender differences” (Figure S1). Additionally, five inclusion criteria were added: (1) full text, (2) clinical trial, (3) meta-analysis, (4) randomized controlled trial, and (4) systematic review, selecting in this way 30 items. Studies related to the use of metformin in combination with other antidiabetic agents have generally been excluded. Instead, several other manuscripts were reviewed according to their relevance to the selected topic through a search in PubMed, Google Scholar, and ResearchGate to find additional relevant items. The benefit–risk profile was studied by analyzing the side effects reported in VigiBase, the WHO database, and EudraVigilance, the European database of ADRs [23,24]. The ratio of cases in women versus men was calculated; a deviated value of more than 20% from 1.0 in both directions (0.8–1.2) indicates the potential impact of sex on the frequency of ADRs [252,253].

## 4. Conclusions

RCT and post-marketing records indicate that metformin is effective and safe in treating hyperglycemia, particularly in type 2 diabetes mellitus. In addition, several clinical studies suggest its use in many other conditions, including polycystic ovary syndrome, weight control in obesity, and as an add-on therapy for various types of cancer. However, there is less evidence for its use in thyroid disorders and neurodegenerative diseases. Despite metformin being on the market for many decades, the interest in validating new clinical uses has not waned, which is surprising. Currently, there are numerous RCTs being developed to verify new applications of metformin. Therefore, new progress can be expected in its clinical use.

The administration of low doses in conjunction with meals can reduce the extent of side effects at the gastrointestinal level, which are generally mitigated gradually in the first two weeks of intake. In order to improve patient compliance and reduce metformin ADRs, it is advised to start with lower doses in all subjects, particularly women, and then gradually increase them to achieve glycemic control. Moreover, monitoring kidney function, particularly in older patients, is recommended.

Existing data on metformin use in real life have not shown significant differences in the antihyperglycemic response between women and men, while adverse drug reactions in women are reported more frequently. A greater care in the choice of posology might decrease the occurrence of side effects in both sexes. It is surprising that women have shown advantages in using metformin to reduce the incidence and mortality of colorectal cancer and improve recovery in COVID-19 compared to men. Additionally, metformin is a drug that has an explicit use, although off-label, in the treatment of polycystic ovary syndrome in women, even at a young age.

In summary, metformin continues to be a drug with satisfactory risk-benefit and cost-benefit ratios, which, by reducing blood glucose, decreases the damage caused by hyperglycemia and is useful in many other diseases related to metabolic disorders.

## Figures and Tables

**Table 1:** Sex-related differences in diabetes mellitus complications and therapeutic use of metformin.

	Female versus Male	References
Diabetes mellitus	Higher all-cause mortality	[122,123]
	Higher risk PAD	[115,116,117]
	Higher RR for oral, stomach, and kidney cancers, and leukemia	[160]
Met: antihyperglycemic effect	No difference	[31]
Met: pharmacokinetics	No difference, considering weight difference and monitoring eGFR.	[76]
Met: ADRs	Higher	[23,24]
Met: CRC-specific mortality *	Lower	[156]
Met: CRC incidence rate *	Lower	[157]
Met: COVID-19 *	Lower severity and mortality	[170,171]

* = Diabetic and/or obese subjects; ADRs = adverse drug reactions; CRC = colorectal cancer; eGFR = estimated glomerular filtration rate; Met = metformin treatment; PAD = peripheral arterial disease; RR = relative risk.

## Data Availability

All data are reported in the text and in the Supplementary Materials.

## Supplementary Materials

The following supporting information can be downloaded at: https://www.mdpi.com/article/10.3390/ph17040478/s1, Figure S1: Flowchart of articles obtained by the PubMed search for metformin up to February 2024; Figure S2: Reports of spontaneous ADRs on metformin obtained by consulting EudraVigilance.
