# Supplementary material for: View on Metformin: Antidiabetic and Pleiotropic Effects, Pharmacokinetics, Side Effects, and Sex-Related Differences
Source: Pharmaceuticals (Basel). 2024 Apr 8;17(4):478. doi: 10.3390/ph17040478 (PMC11054066; doi:10.3390/ph17040478)
Supplement: Supplementary file 1 [file pharmaceuticals-17-00478-s001.zip › pharmaceuticals-2936996-supplementary.pdf]

Supplementary Materials

View on Metformin: Antidiabetic and Pleiotropic Effects, Pharmacokinetics, Side Effects and Sex-Related Differences

Guglielmina Froldi

Department of Pharmaceutical and Pharmacological Sciences, University of Padova, 35131 Padova, Italy; g.froldi@unipd.it; Tel.: +39 049 8275092

Abstract: Metformin is a synthetic biguanide used as an antidiabetic drug in type 2 diabetes mellitus, achieved by studying the bioactive metabolites of *Galega officinalis* L. It is also used off-label in various other diseases, such as subclinical diabetes, obesity, polycystic ovary syndrome, etc. In addition, metformin is proposed as an add-on therapy for several conditions, including autoimmune diseases, neurodegenerative diseases, and cancer. Although metformin has been used for many decades, it is still the subject of many pharmacodynamic and pharmacokinetic studies in light of its extensive use. Metformin acts at mitochondrial level by inhibiting the respiratory chain, thus increasing the AMP/ATP ratio and, subsequently, activating the AMP-activated protein kinase. However, several other mechanisms have been proposed, including binding to presenilin enhancer 2, increasing GLP-1 release, and modification of microRNA expression. Regarding its pharmacokinetics, after oral administration, metformin is absorbed, distributed, and eliminated, mainly through the renal route, using transporters for cationic solutes, since it exists as an ionic molecule at physiological pH. In this review, particular consideration has been paid to literature data from the last 10 years, deepening the study of clinical trials inherent to new uses of metformin, the differences in effectiveness and safety observed between the sexes, and the unwanted side effects. For this last objective, metformin safety was also evaluated using both EudraVigilance and VigiBase, respectively, the European and WHO databases of the reported adverse drug reactions, to assess the extent of metformin side effects in the real-life use.

Keywords: AMPK; miRNA; antidiabetic drugs; ADRs; sex-differences; ADMET; pharmacovigilance; T2DM

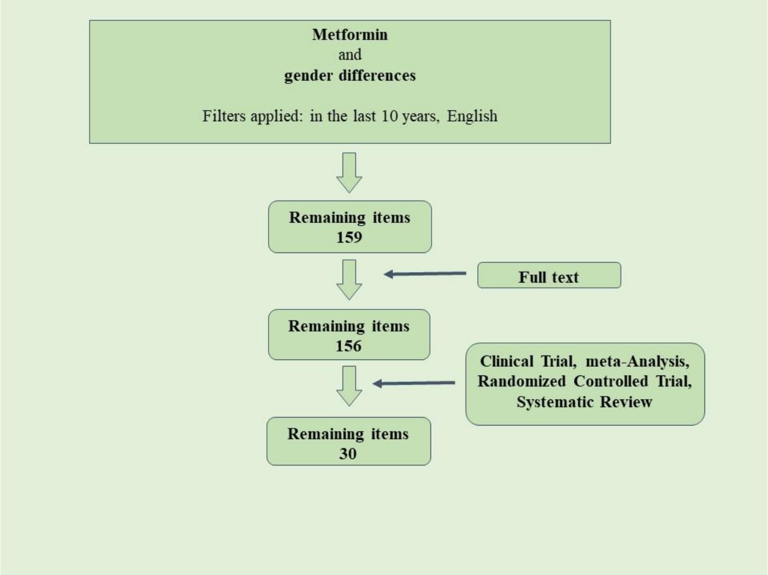

Figure S1: Flowchart of articles obtained by the PubMed search for metformin up to February 2024.

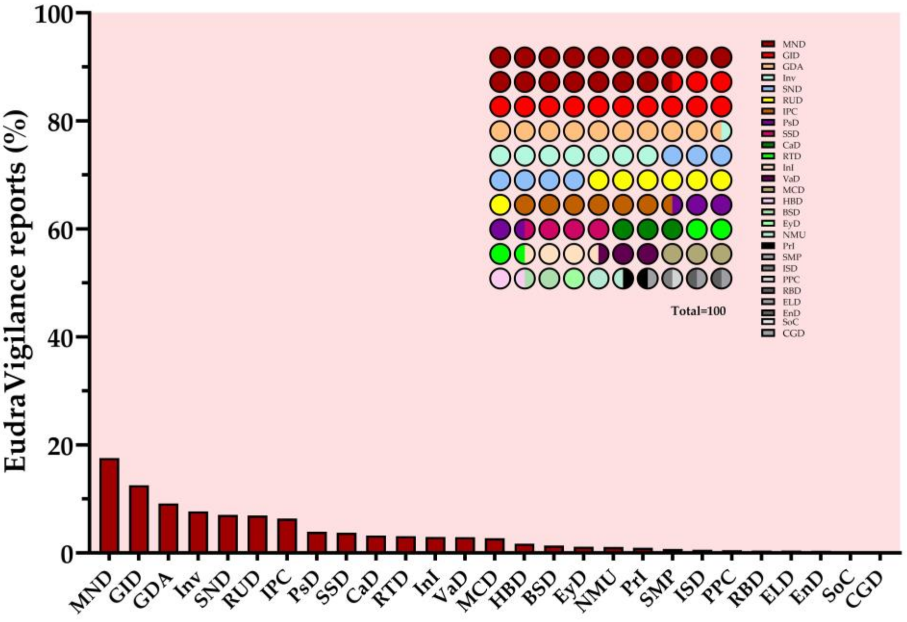

Figure S2: Reports of spontaneous ADRs on metformin obtained by consulting EudraVigilance.

Disclaimer/Publisher's Note: The statements, opinions and data contained in all publications are solely those of the individual author(s) and contributor(s) and not of MDPI and/or the editor(s). MDPI and/or the editor(s) disclaim responsibility for any injury to people or property resulting from any ideas, methods, instructions or products referred to in the content.
